# Distinct Roles of N-Terminal Fatty Acid Acylation of the Salinity-Sensor Protein SOS3

**DOI:** 10.3389/fpls.2021.691124

**Published:** 2021-09-23

**Authors:** Irene Villalta, Elena García, Dámaso Hornero-Mendez, Raúl Carranco, Carlos Tello, Imelda Mendoza, Anna De Luca, Zaida Andrés, Karin Schumacher, José M. Pardo, Francisco J. Quintero

**Affiliations:** ^1^Institut de Recherche sur la Biologie de l’Insecte, Université de Tours, Tours, France; ^2^Institute of Plant Biochemistry and Photosynthesis, Consejo Superior de Investigaciones Cientificas and Universidad de Sevilla, Seville, Spain; ^3^Instituto de la Grasa, Consejo Superior de Investigaciones Científicas, Seville, Spain; ^4^SelfDecode, Miami, FL, United States; ^5^Centre for Organismal Studies, Universität Heidelberg, Heidelberg, Germany

**Keywords:** protein acylation, salinity tolerance, nuclear entry, salt-overly-sensitive pathway, SOS3/CBL4

## Abstract

The Salt-Overly-Sensitive (SOS) pathway controls the net uptake of sodium by roots and the xylematic transfer to shoots in vascular plants. SOS3/CBL4 is a core component of the SOS pathway that senses calcium signaling of salinity stress to activate and recruit the protein kinase SOS2/CIPK24 to the plasma membrane to trigger sodium efflux by the Na/H exchanger SOS1/NHX7. However, despite the well-established function of SOS3 at the plasma membrane, SOS3 displays a nucleo-cytoplasmic distribution whose physiological meaning is not understood. Here, we show that the N-terminal part of SOS3 encodes structural information for dual acylation with myristic and palmitic fatty acids, each of which commands a different location and function of SOS3. *N*-myristoylation at glycine-2 is essential for plasma membrane association and recruiting SOS2 to activate SOS1, whereas *S*-acylation at cysteine-3 redirects SOS3 toward the nucleus. Moreover, a poly-lysine track in positions 7–11 that is unique to SOS3 among other *Arabidopsis* CBLs appears to be essential for the correct positioning of the SOS2-SOS3 complex at the plasma membrane for the activation of SOS1. The nuclear-localized SOS3 protein had limited bearing on the salt tolerance of *Arabidopsis*. These results are evidence of a novel *S*-acylation dependent nuclear trafficking mechanism that contrasts with alternative subcellular targeting of other CBLs by *S*-acylation.

## Introduction

The addition of fatty acids to proteins through acyl linkages, known as protein acylation, is a ubiquitous eukaryotic post-translational modification serving to control protein trafficking and biological function (Aicart-Ramos et al., 2011; Turnbull and Hemsley, 2017). The most prominent types of fatty acid acylations are myristoylation and palmitoylation. Protein *N*-myristoylation is a cotranslational lipidic modification. The terminal methionine is removed from the nascent polypeptide by methionyl-aminopeptidases to expose the acceptor glycine to the covalent attachment of myristic acid by *N*-myristoyltransferases (NMTs). *N*-myristoylation directs proteins to varied intracellular destinations and mediates protein–protein interactions. The lipid moiety attachment results in increased hydrophobicity that may trigger or not membrane anchorage since *N*-myristoylation is often necessary but not sufficient for stable membrane association. *N*-myristoylation usually occurs together with the *S*-acylation of proximal cysteine residues (Castrec et al., 2018), which may enhance hydrophobicity enough to enforce membrane association. *S*-acylation, also known as protein palmitoylation, is a reversible protein acylation catalyzed by the membrane-integral palmitoyl-acyl-transferases (PATs; Philippe and Jenkins, 2019). In *S*-acylation, a chain fatty acid, usually palmitic, covalently attaches to a cysteine residue *via* a thioester bond. A critical difference between these two protein acylations with fatty acids is that, whereas protein *N*-myristoylation is largely irreversible, *S*-acylation is a fully reversible post-translational modification, akin to protein phosphorylation, through the interplay of PATs and acyl-protein thioesterases (APTs; Hemsley, 2020). There is no clear consensus motif for the *in silico* identification of *S*-acylated proteins or *S*-acylation sites within a protein, the only exception being that a cysteine residue at position 3 next to a glycine in position 2 is often found to be associated with concurrent *N*-myristoylation and *S*-palmitoylation (Hemsley et al., 2013; Castrec et al., 2018).

Knowledge on protein *S*-acylation and its consequences on protein function and trafficking is lagging behind that of myristoylation because of the recent discovery of PATs and the limited technical resources available to study *S*-acylation (Li and Qi, 2017; Hemsley, 2020). The roles of *S*-acylation are still poorly understood, but they go certainly beyond the regulation of membrane association. For instance, several components of the cellulose synthase complex are integral membrane proteins with several transmembrane domains. Yet, the CseA protein undergoes multiple *S*-acylations, and mutation of the *S*-acylation sites renders the whole cellulose synthase complex inactive and trapped in Golgi vesicles unable to fuse with the plasma membrane (Kumar et al., 2016). The asymmetric distribution of the receptor kinase SCHENGEN1 (SGN1), critical for the proper positioning of the Casparian strip in the root endodermis, is maintained by cycles of *S*-acylation and de-*S*-acylation (Alassimone et al., 2016).

The Calcineurin B-Like (CBL) proteins are calcium sensors that regulate the activity and subcellular localization of CBL-Interacting Protein Kinases (CIPKs). The subcellular targeting of CBLs and the corresponding CBL-CIPK complexes are largely controlled by CBL acylation with the myristic and palmitic fatty acids. *S*-acylation of CBL2, CBL3, CBL6, and CBL10 by the tonoplast-localized PAT10 mediates their association with vacuoles, where they regulate processes that are important for V-ATPase activity, vacuolar morphology, Mg^2+^ compartmentation, polarized growth of pollen tubes, seed development, ABA-mediated stomatal movements, and stress responses (Batistic et al., 2012; Tang et al., 2012, 2015; Zhou et al., 2013; Eckert et al., 2014; Pandey et al., 2015; Steinhorst et al., 2015; Zhang et al., 2017; Feng et al., 2018; Song et al., 2018; Chai et al., 2019). *N*-terminal myristoylation targets CBL1 to the endoplasmic reticulum (ER) and subsequent *S*-acylation facilitates further trafficking toward the plasma membrane (Batistic et al., 2008). Dual-acylated CBL1 together with CIPK23 activates the K^+^-uptake proteins AKT1 and HAK5 (Xu et al., 2006; Ragel et al., 2015), inhibits ammonium transport (Straub et al., 2017), and opens the anion channels SLAC1 and SLAH3 (Maierhofer et al., 2014). In an alternative complex with CIPK26, CBL1 regulates the *Arabidopsis* NADPH oxidase RBOHF (Drerup et al., 2013).

The highly conserved Salt-Overly-Sensitive (SOS) pathway is a critical determinant of salt tolerance in various plant species (Ji et al., 2013), including important crops such as tomato and rice (Martinez-Atienza et al., 2007; Olias et al., 2009; El Mahi et al., 2019). The core components of the SOS pathway in *Arabidopsis thaliana* are the plasma membrane Na^+^/H^+^ antiporter SOS1/NHX7, its activating kinase SOS2/CIPK24, and the Ca^2+^-sensing proteins SOS3/CBL4 and SCaBP8/CBL10 (Qiu et al., 2002; Quintero et al., 2002; Quan et al., 2007). Both SOS3/CBL4 and SCaBP8/CBL10 activate and recruit SOS2/CIPK24 to the plasma membrane for the activation of SOS1, but whereas SOS3/CBL4 is preferentially expressed in roots and determines the salt tolerance of this organ, SCaBP8/CBL10 activity is more prevalent in shoots (Liu and Zhu, 1998; Quintero et al., 2002; Guo et al., 2004; Quan et al., 2007).

Dual lipid acylation of SOS3 by myristoylation and palmitoylation has been reported (Ishitani et al., 2000; Batistic et al., 2008; Held et al., 2011). *N*-myristoylation at Gly2 is essential for SOS3 function in the salt tolerance of *Arabidopsis* (Ishitani et al., 2000), but the mechanism involved remains uncertain because the myristoylation status of SOS3 did not significantly affect the association of SOS3 to the plasma membrane where SOS1 operates (Ishitani et al., 2000; Shi et al., 2002). Conversely, SOS3 recruits SOS2 to the plasma membrane (Quintero et al., 2002), but the role of myristoylation in this process is not yet determined. On the other hand, the *S*-acylation of SOS3 appears to be important for the trafficking of protein complexes in which SOS3 participates (Held et al., 2011). SOS3/CBL4, together with the interacting kinase CIPK6, facilitates the trafficking of the K^+^-selective channel AKT2 to the plasma membrane and its activation thereafter. Notably, both *N*-myristoylation and *S*-acylation of SOS3/CLB4 were required to exit the AKT2/CIPK6/CBL4 complex of the ER and efficient targeting of the channel to the plasma membrane *via* a novel BFA-insensitive pathway (Held et al., 2011). However, the role of *S*-acylation of SOS3 in the operation of the SOS pathway for salt tolerance has not been assessed.

In this work, we aimed to determine the precise role of dual fatty acylation in the function of SOS3 in the SOS pathway using a reconstituted SOS system in yeast cells. This approach allows the accurate dissection of each of the SOS pathway constituents in a simplified cellular system (Quintero et al., 2002, 2011). Results in yeast, together with the analysis of protein localization and function *in planta*, showed that *N*-myristoylation of SOS3 was essential for the attachment of plasma membrane, recruitment of SOS2, and the activation of SOS1. *S*-acylation was partially dispensable in this regard. Notably, the nuclear localization SOS3 required *S*-acylation of SOS3 at Cys3 since its mutation to Ala abrogated nuclear entry. However, preventing both *N*-myristoylation and *S*-acylation of SOS3 restored nuclear localization, suggesting that the *S*-acylation of *N*-myristoylated SOS3 redirects protein trafficking from plasma membrane to the nucleus and that the SOS3 polypeptide contains intrinsic information for nuclear entry. Furthermore, we have identified a poly-lysine track adjacent to the acylation sites as a relevant structural determinant for the proper localization of the SOS2-SOS3 complex in the membrane plane for the effective activation of the target protein SOS1.

## Results

**Figure 1:**
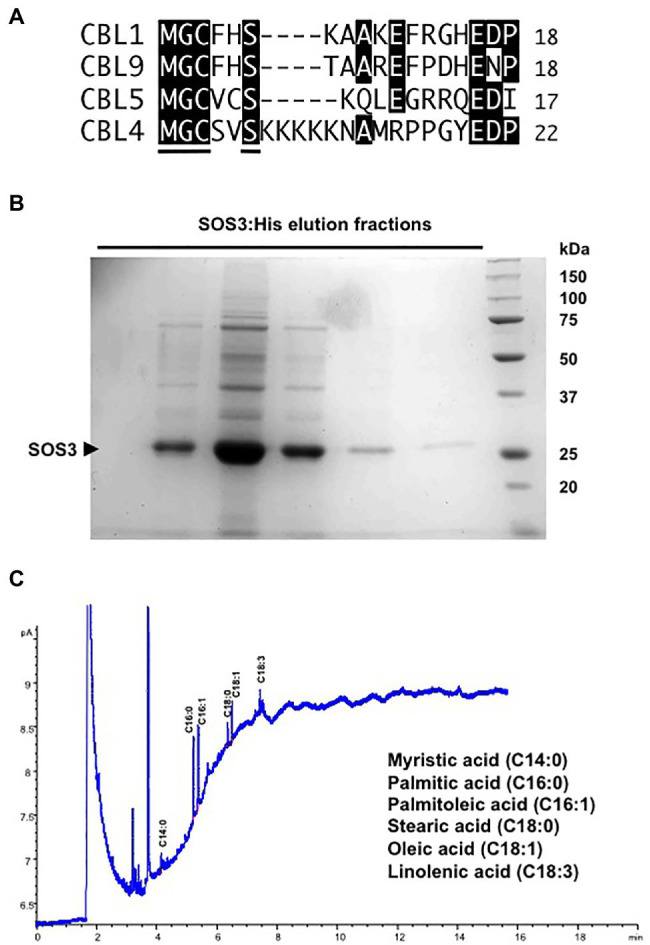
Fatty acid acylations of SOS3. **(A)** Alignment of the N-terminal sequences of CBL1, CBL4/SOS3, CLB5, and CBL9 that undergo N-myristoylation and S-acylation in *Arabidopsis*. The consensus motif for dual acylation (MGCxxS) is underlined in the CBL4/SOS3 sequence. **(B)** His-tagged SOS3 protein expressed in yeast and purified by Ni-NTA affinity chromatography. Shown are the elution fractions with 250mM imidazole resolved by SDS-PAGE and stained with Coomassie. The arrowhead indicates the SOS3 band. **(C)** Profile of fatty acids attached to purified SOS3 protein. Peaks in the GC chromatogram corresponding to myristic (C14:0), palmitic (C16:0), palmitoleic (C16:1), stearic (C18:0), oleic (C18:1), and linolenic (C18:3) acids are shown. Fatty acids were identified by comparison with known standards.

SOS3 contains an amino-terminal consensus motif (MGCxxS) for adjacent *N*-myristoylation and *S*-acylation (Figure 1A). *N*-myristoylation at Gly2 is essential for the function of SOS3 in salt tolerance (Ishitani et al., 2000), whereas *S*-acylation of SOS3 has been linked to the trafficking of protein complexes (Held et al., 2011). Since protein acylation with fatty acids is highly conserved in eukaryotes, we first confirmed that SOS3 is myristoylated and *S*-acylated in yeast cells and then tested whether *N*-myristoylation and *S*-acylation of SOS3 influenced protein localization and the operability of the SOS pathway in a reconstituted SOS pathway in yeast (Quintero et al., 2002).

To determine the profile of fatty acid acylation, His-tagged SOS3 was expressed in yeast cells and recovered by affinity purification chromatography with Ni-NTA resin (Figure 1B). Next, the protein-linked fatty acids in the highly purified SOS3 sample were determined by gas chromatography (Figure 1C). Results confirmed that SOS3 underwent myristoylation and *S*-acylation with an array of fatty acids that primarily consisted of palmitic (C16:0) and palmitoleic (C16:1) acids, and smaller amounts of stearic (C18:0), oleic (C18:1), and linolenic (C18:3) acids. Heterogeneity in the fatty acids that are naturally attached with proteins *in vivo* by palmitoyl transferases has been described (Aicart-Ramos et al., 2011). The low myristate signal relative to *S*-linked fatty acids likely reflected the greater lability of thioester bonds relative to the stable amide linkage of myristate to the terminal glycine (Resh, 2016).

**Figure 2:**
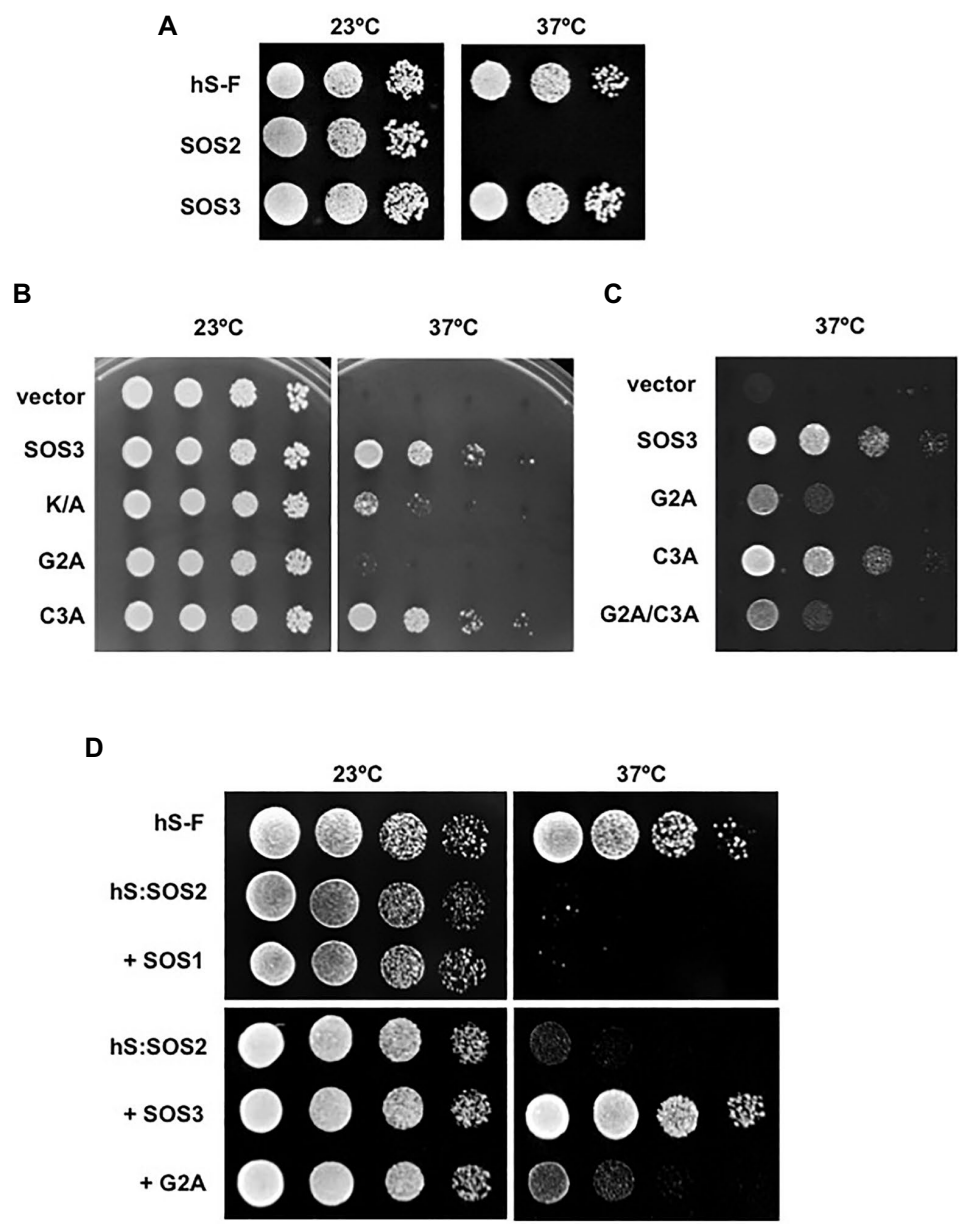
Recruitment of SOS proteins to the plasma membrane. **(A)** SOS2 and SOS3 were fused with the hSos reporter protein and were expressed in the yeast strain *cdc25-2*. Decimal dilutions were spotted onto duplicated selective plates and incubated at 23°C or 37°C. Growth at 37°C indicates recruitment to the plasma membrane. Plasmid pADNS–SosF encoding a hSos protein fused to Ras farnesylation signal (hS-F) was used as a positive control. **(B)** Wild-type SOS3 and mutants K/A, G2A, and C3A were fused with the reporter hSos and processed as in (A). Growth a 37°C indicates attachment to the plasma membrane. **(C)** Similar assay to that in (B) but using 10-fold higher culture densities for the drop-spots to compare the single SOS3 mutants G2A and C3A to the double mutant G2A/C3A. **(D)** SOS2 fused to the reporter hSos (hS:SOS2) was co-expressed with SOS1, wild-type SOS3, and the SOS3 mutant G2A. Samples were processed as in (A). Growth at 37°C indicates the recruitment of the hSos:SOS2 chimera to the plasma membrane. Farnesilated hSos protein (hS-F) was used as a positive control.

To investigate the precise function of *N*-myristoylation and *S*-acylation of SOS3, residues Gly2 and Cys3 were mutated to alanine, either separately or combined (henceforth, G2A and C3A mutations). First, we addressed whether *N*-myristoylation and *S*-acylation influenced the binding of SOS3 to the plasma membrane or not using the hSos-Recruitment System (SRS). This method monitors the targeting to the plasma membrane of a protein fused to a truncated form of the human protein hSos (*Son of Sevenless*), which is the functional homolog of the yeast Ras guanyl nucleotide exchange factor CDC25. These proteins are interchangeable in yeast and must be recruited to the cell membrane to allow the progression of the cell cycle of a *cdc25* mutant. Recruitment of hSos to the plasma membrane of the thermosensitive yeast mutant *cdc25-2* restores growth at the restrictive temperature of 37°C (Aronheim, 1997). In this assay, the fusion of wild-type SOS3 to the reporter protein hSos restored the growth of *cdc25-2* cells at 37°C, indicating the effective recruitment to the plasma membrane, whereas the SOS2-hSos fusion protein failed to complement the growth defect of the *cdc25-2* mutant (Figure 2A). The non-myristoylatable mutant SOS3-G2A also failed to restore the growth (Figure 2B). Together, these results indicate that the efficient targeting of SOS3 to the plasma membrane requires *N*-myristoylation at Gly2. By contrast, the mutation of *S*-acylation site at Cys3 (C3A mutation) had little effect on the targeting of SOS3 to the plasma membrane, both alone and in combination with the G2A mutation (Figures 2B,C). Plating conditions designed to monitor small changes in the attachment of SOS3 protein to the plasma membrane indicated that *S*-acylation had no significant contribution to membrane association because the growth of the single mutant G2A and double mutant G2A/C3A was indistinguishable (Figure 2C).

The *N*-terminal of SOS3 contains a poly-lysine track (poly-K henceforth) that is unique to SOS3 when compared with the other nine CBLs encoded in the *Arabidopsis* genome (Figure 1A). Poly-basic tracks can facilitate the anchorage of peripheral proteins to cellular membranes (Monné et al., 1998; Killian and von Heijne, 2000). Replacement of the poly-K with poly-A (K/A henceforth) reduced but did not abrogate the ability of SOS3 to bind to the plasma membrane as reported by the SRS test (Figure 2B).

Next, we tested whether the *N*-myristoylation of SOS3 was required for the recruitment of SOS2 to the plasma membrane. For this experiment, SOS2 was fused with the reporter protein hSos. Growth tests at restrictive temperature showed that SOS2 *per se* failed to bind to the plasma membrane with and without the co-expression of its target protein SOS1 (Figure 2D). However, hSos-tagged SOS2 was efficiently recruited to the plasma membrane when co-expressed with SOS3 but not with the non-myristoylatable mutant SOS3-G2A (Figure 2D). These results confirm that SOS3 recruits SOS2 to the plasma membrane and demonstrate that the *N*-myristoylation of SOS3 is essential for this function.

**Figure 3:**
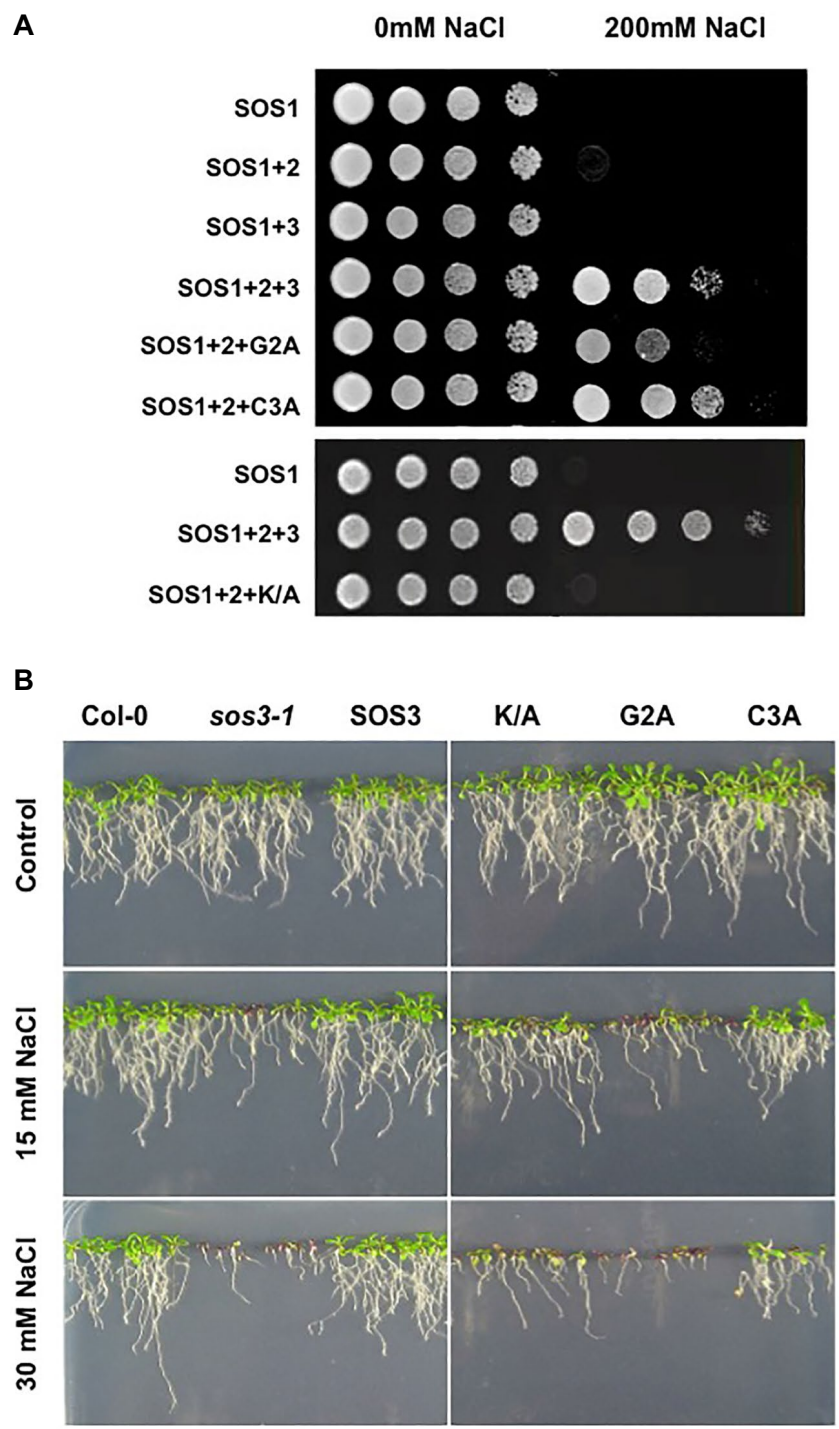
Functionality of the SOS pathway. **(A)** Reconstitution of the SOS pathway in yeast. YP890 cells expressing the indicated combination of Arabidopsis SOS1, SOS2, and SOS3 proteins were grown overnight in liquid AP medium with 1mM KCl. Five microliters of serial decimal dilutions were spotted onto plates of the same medium or supplemented with 200mM NaCl. Plates were incubated at 28°C and photographed after 4days. G2A, C3A and K/A denote the corresponding SOS3 mutants. **(B)** Ten-day old seedlings of homozygous transgenic lines of the *sos3-1* mutant of *Arabidopsis* transformed to express the wild-type SOS3 protein (SOS3) or the K/A, G2A, and C3A mutants grown in MS plates were transferred to LAK medium with 1mM KCl and supplemented or not with 15mM and 30mM NaCl. Col-0 and the *sos3-1* mutant were used as control. Plates were incubated vertically for 12–14days at 25°C with a 16h/8h light period.

We then studied the consequences of *N*-myristoylation and *S*-acylation of SOS3 on the activity of a reconstituted SOS system in the yeast cell (Quintero et al., 2002). For this, SOS1, SOS2, and SOS3 in various combinations were expressed in the YP890 yeast strain, in which the genes encoding the primary Na^+^ transporters at the plasma membrane (Na^+^, K^+^-ATPases ENA1 to ENA4, and the Na^+^/H^+^ antiporter NHA1) and endosomes (Na^+^, K^+^/H^+^ antiporter NHX1) have been deleted and the SOS1 protein of *Arabidopsis* is expressed from a chromosomal insertion (Guo et al., 2004). Only the co-expression of SOS1, SOS2, and SOS3 restored salt tolerance in YP890 cells (Figure 3A). As expected from the defective recruitment of SOS2-SOS3 complex to the plasma membrane, the substitution of the wild-type SOS3 protein with the SOS3-G2A mutant significantly reduced the salt tolerance imparted by the SOS proteins. *S*-acylation of SOS3 at Cys3 was dispensable for the operability of the SOS pathway in this heterologous system. However, the double mutant protein G2A/C3A was less effective than the G2A single mutant, suggesting that *S*-acylation may sustain some degree of membrane attachment of SOS3 when the myristoyl group is absent. We have shown elsewhere that the *S*-acylation of SOS3 at Cys3 can proceed without prior *N*-myristoylation at Gly2 *in planta* (Park et al., 2021). Thus, the partial efficacy of the SOS3-G2A mutant to restore salt tolerance to yeast cells suggests some capacity for the assembly of the SOS protein complex facilitated by *S*-acylation. In the yeast system, the K/A mutant of SOS3 also failed to reconstitute a fully functional SOS pathway (Figure 3A).

To validate these results *in planta*, the wild-type SOS3 and mutants G2A, C3A and K/A were tested for the subcellular distribution and complementation of the salt-sensitivity of the *sos3-1* mutant. The cDNAs encoding the wild-type SOS3 and the G2A, C3A, and K/A variants were functionally linked to the *35S* promoter for the transformation of the *sos3-1* mutant of *Arabidopsis*, and homozygous T3 transgenic plants were tested for salt tolerance. The C3A mutant partly restored the salt tolerance of transformed *sos3-1* plants, whereas the G2A mutant could not complement at all (Figure 3B). Notably, the SOS3-K/A mutant largely failed to suppress the salt-sensitivity of *sos3-1*. Together these results confirm the essentiality of *N*-myristoylation of SOS3 in the salt tolerance mediated by the SOS1-SOS2-SOS3 module (Ishitani et al., 2000) and suggest that the *S*-acylation of SOS3 plays an accessory role in this physiological output.

**Figure 4:**
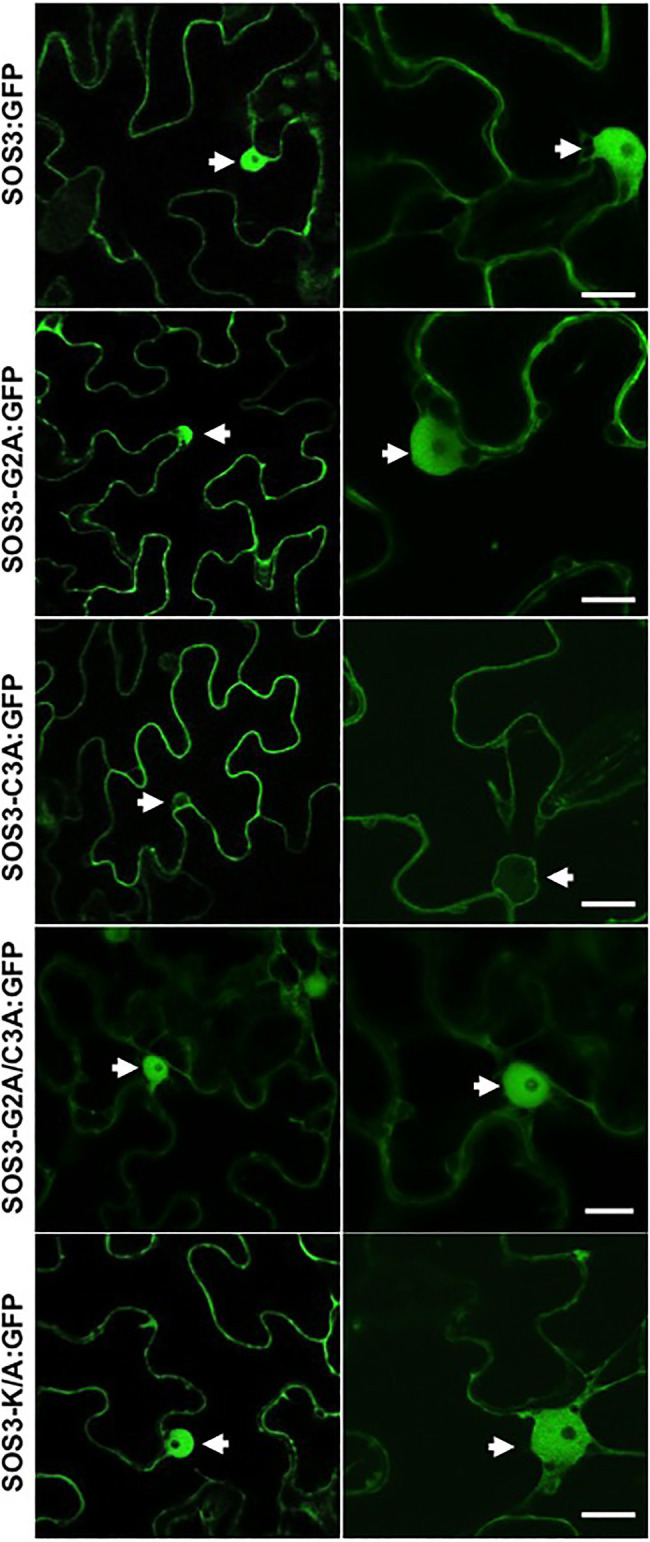
Subcellular localization of SOS3 and mutants. The subcellular distribution of wild-type SOS3 and mutants G2A, C3A, G2A/C3A and K/A, all fused to GFP, was tested by transient expression in *Nicotiana benthamiana*. Left and right side panels are micrographs taken at different magnifications. Arrows indicate nuclei. Scale bars=10μm.

**Figure 5:**
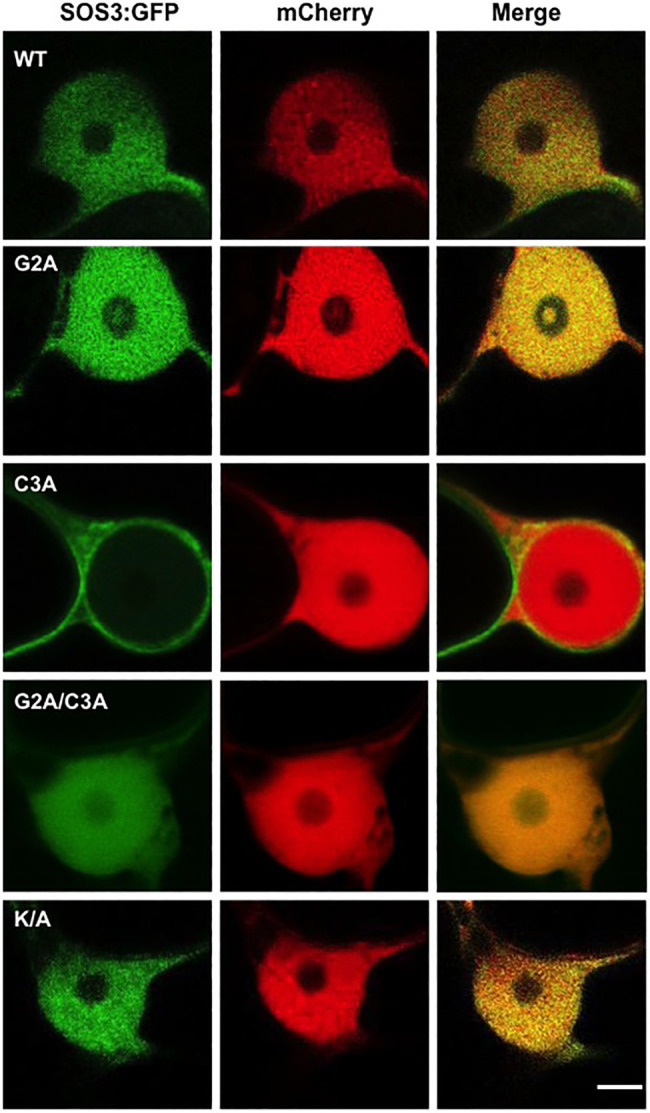
*S*-acylation directs SOS3 nuclear entry. Nuclear entry or exclusion of wild-type SOS3 and mutants proteins G2A, C3A, G2A/C3A, and K/A, all fused to GFP, as shown by transient expression in *N. benthamiana*. The red-fluorescent protein mCherry was used as a control for nuclear localization. Scale bar=3μm.

The cDNAs encoding the SOS3 variants (wild-type and mutants G2A, C3A, G2A/C3A, and K/A) were fused with GFP and transiently expressed in *Nicotiana* to test protein localization. As previously reported (Batistic et al., 2010), SOS3-GFP showed a nucleo-cytoplasmic distribution (Figure 4). Notably, the C3A mutation affecting the *S*-acylation of SOS3 suppressed the nuclear localization of SOS3 completely, as confirmed by the co-expression of free mCherry protein that naturally accumulates in nuclei (Figure 5). Neither the G2A nor K/A mutations affected the nuclear entry of SOS3 (Figures 4, 5). Surprisingly, the double mutation G2A/C3A restored nuclear localization of the non-acylated protein.

**Figure 6:**
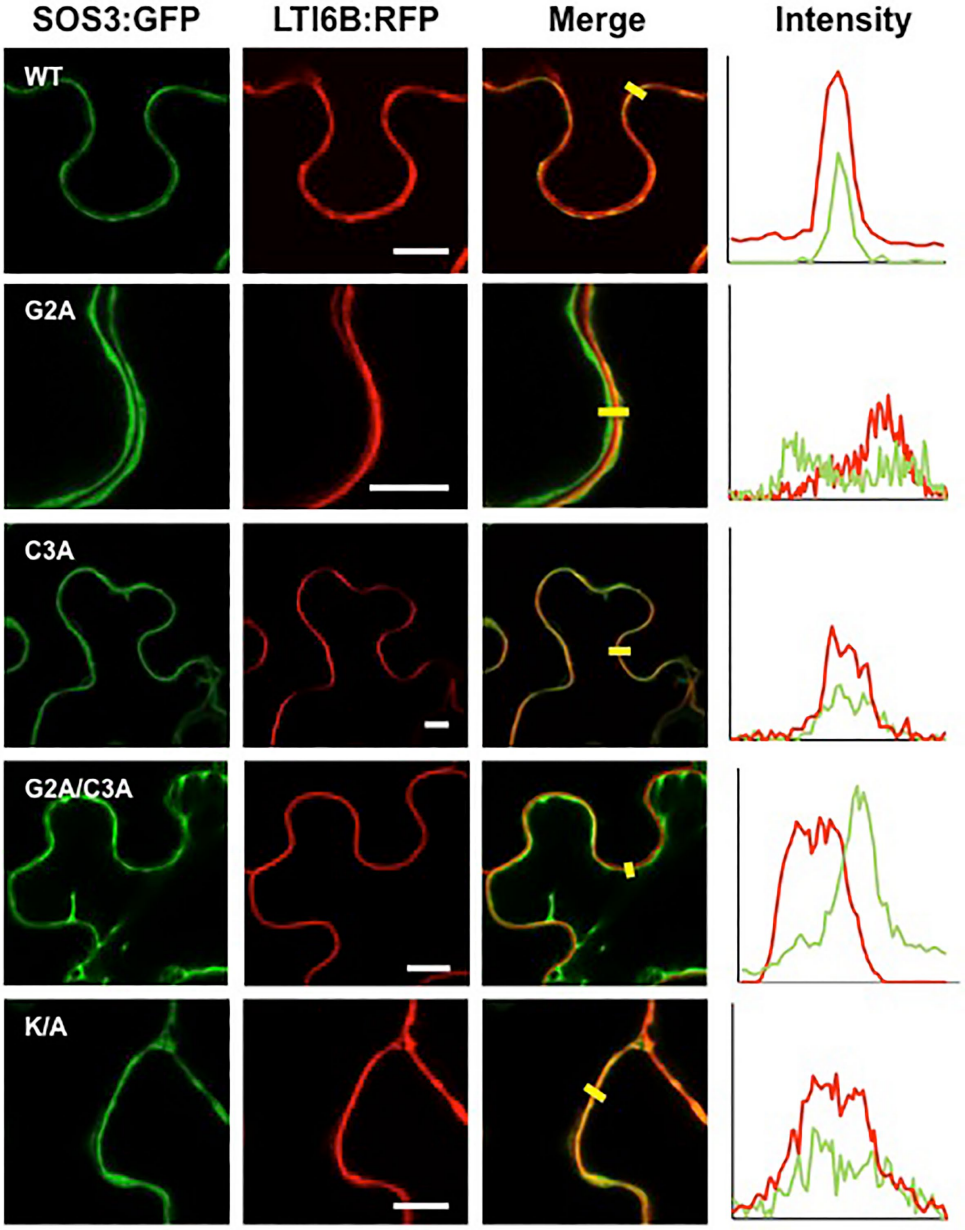
Plasma membrane attachment of SOS3 mutant proteins. Wild-type SOS3 and mutant proteins G2A, C3A, G2A/C3A, and K/A, all fused to GFP, were transiently co-expressed in *N. benthamiana* with the plasma membrane marker LTI6B:RFP. The plots on the right show the fluorescence intensity in the green and red channels along transects depicted as yellow bars in the micrographs with green and red channels merged. The coincidence of maxima in green and red traces indicates protein co-localization. White bars in middle column=10μm.

**Figure 7:**
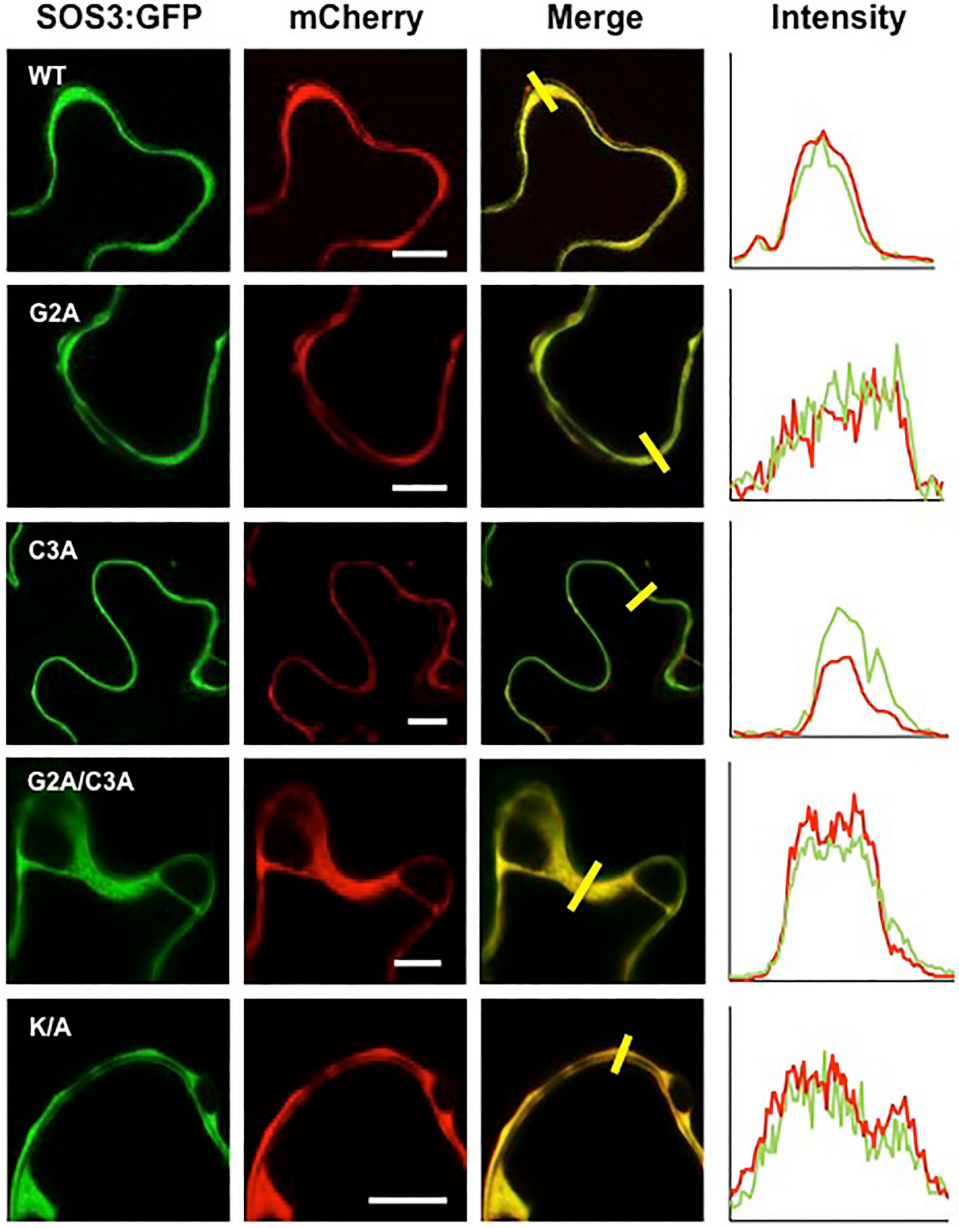
Cytoplasmic localization of SOS3 mutant proteins. Wild-type SOS3 and mutant proteins G2A, C3A, G2A/C3A, and K/A, all fused with GFP, were transiently co-expressed in *N. benthamiana* with the nucleo-cytoplasmic marker mCherry. The plot on the right shows the fluorescence intensity in the green and red channels along transects depicted as yellow bars in the micrographs with green and red channels merged. The coincidence of maxima in green and red traces indicates protein co-localization. White bars in middle column=10μm.

Co-expression of SOS3 variants with the nucleocytoplasmic protein mCherry and the plasma membrane marker LTI6B-RFP, followed by analysis of fluorescence intensity transects in the green and red channels, showed that the plasma membrane association of SOS3 was lost in the G2A mutant, which became cytosolic as the free mCherry (Figures 6, 7). In this assay, mutations C3A and K/A had no substantial effect on plasma membrane binding since their peak fluorescence overlapped with that of LTI6B-RFP in a transect across the cell surface, whereas the double mutant G2A/C3A lost the attachment to the plasma membrane (Figures 6, 7).

**Figure 8:**
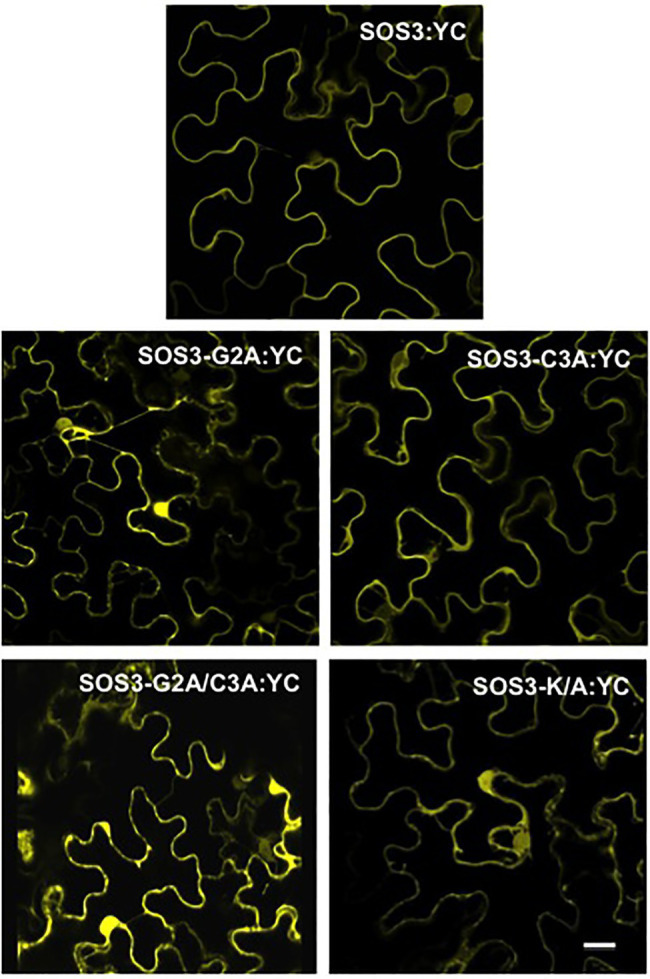
Interaction of SOS2 with SOS3 protein variants by BiFC. Wild-type SOS3 and mutants G2A, C3A, G2A/C3A, and K/A were C-terminally fused with the C-terminal half of YFP (YC) and individually co-expressed in *N. benthamiana* together with SOS2 N-terminally fused with the N-terminal half of YFP. Reconstituted YFP fluorescence was determined by confocal microscopy 3days after agroinfection. Co-transformation controls of SOS3-YC and YN-SOS2 with the reciprocal empty vectors produced blank images. Scale bar=20μm.

The results obtained regarding the K/A mutation were somehow contradictory. The SOS3-K/A protein was not efficiently targeted to the yeast’s plasma membrane and failed to reconstitute a functional SOS module (Figures 1, 2). However, transient expression in tobacco showed no significant decrement of plasma membrane binding, yet the SOS3-K/A protein could not complement the *sos3-1* mutant (Figures 3, 4). To investigate further the properties of the SOS3 mutant variants, we inspected their ability to interact with and recruit the kinase partner SOS2 to the plasma membrane *in planta*. The translational fusion YN-SOS2 was co-expressed in *Nicotiana* leaves with wild-type SOS3-YC and equivalent constructs with mutants G2A, C3A, K/A, and the double mutant G2A/C3A. The BiFC assays of SOS2 with the SOS3 variants indicated that all SOS3 mutants retained their capacity to interact with SOS2 albeit with distinct properties (Figure 8). Whereas the wild-type SOS2/SOS3 complex was essentially, if not exclusively, located to the plasma membrane, the complexes formed by SOS2 with either SOS3-G2A, SOS3-G2A/C3A, and SOS3-K/A showed a marked cytoplasmic localization. The complex of SOS2 with the SOS3-C3A protein was excluded from the nucleus, which is consistent with the behavior of SOS-C3A alone. However, the complex of SOS2 with the double mutant SOS3-G2A/C3A could be observed in nuclei, in agreement with the nuclear localization of SOS3-G2A/C3A expressed alone.

## Discussion

Here, we provide evidence supporting that the *N*-terminal part of SOS3 encodes rich information that determines the subcellular distribution of SOS3 and its biological function. In short, we show that *N*-myristoylation of SOS3 at Gly2 is required for the recruitment of SOS2 to the plasma membrane and the activation of SOS1 for salt tolerance. Moreover, the unique poly-lysine domain adjacent to the acylation sites plays a potential role in the correct positioning of SOS3 at the plasma membrane for the effective activation of SOS1. Surprisingly, *S*-acylation at Cys3, instead of reinforcing the membrane association of SOS3, served to redirect the protein toward the nucleus. The latter result suggests the existence of a palmitoylation-dependent pathway for protein trafficking to the nucleus.

Previous work had demonstrated that SOS3 is *N*-myristoylated at Gly2 and that this post-translational modification was essential for the role of SOS3 in the salt tolerance of *Arabidopsis* (Ishitani et al., 2000). However, the precise role of *N*-myristoylation could not be determined because no significant difference in membrane association between the myristoylated and non-myristoylated SOS3 could be resolved. Here, we have shown by two different approaches in yeast and tobacco that *N*-myristoylation at Gly2 is essential for the attachment of SOS3 to the plasma membrane, which is a requisite for effective activation of SOS1 and salt tolerance. However, we also show that *N*-myristoylation is only one of the at least two structural determinants mapped at the amino-terminus of SOS3 with functional significance in membrane anchorage and activation of the SOS pathway. SOS3 presents a track of five lysine residues (poly-K) at the N-terminal end that is unique among the *Arabidopsis* CBLs (Figure 1A). While fatty acids anchor proteins to the membrane, basic amino acids can interact with the negatively charged hydrophilic heads of membrane phospholipids and strengthen the interaction (Monné et al., 1998; Killian and von Heijne, 2000). For instance, small GTPases of plants attach to cell membranes by post-translational prenylation and *S*-acylation. Deleting a poly-basic domain proximal to the *S*-acylation site disrupted association with the plasma membrane and solubilized the mutant protein (Lavy and Yalovsky, 2006). Here, our SRS assay in yeast indicated that the poly-K track of SOS3 adjacent to the double-lipidation motif was strictly needed for the productive anchorage of SOS3 to the plasma membrane after *N*-myristoylation at Gly2. Although we have not ruled out experimentally that mutating the poly-K track interfered with the *N*-myristoylation of SOS3, this was unlikely to happen. Most proteins targeted for co-translational *N*-myristoylation have an N-terminal consensus sequence of MGxxx(S/T) within the six residues that fit into the substrate-binding pocket of NMTs (Maurer-Stroh et al., 2002; Castrec et al., 2018). Only the N-terminal hexapeptide determines substrate specificity by NMTs in *N*-myristoylation. There are no well-defined requirements for residues in positions 7–11, except that relatively small and polar side-chains are preferred (Maurer-Stroh et al., 2002; Castrec et al., 2018). The corresponding sequence in SOS3 is MGCSVSKKKKK. Substitution of the poly-K with poly-A in mutant SOS3-K/A would still fully match the consensus for N-terminal myristoylation without adding bulky or negative residues that could interfere with binding by NMTs (Castrec et al., 2018). Moreover, CBL1, CBL5, and CBL9 are also subjected to *N*-myristoylation, and neither of these proteins displays the SOS3-specific poly-K track. In fact, CBL1 and CBL9 both have alanine residues at positions 8 and 9, corresponding to the poly-K track in SOS3/CBL4 (positions 7–11; Figure 1A). Together, these data strongly suggest that the SOS3-K/A mutant is being *N*-myristoylated and that the poly-K track is a structural feature that specifically facilitates membrane association and the biological function of SOS3 in salt tolerance. The possibility that not all nascent SOS3 polypeptides are *N*-myristoylated during synthesis (see below), together with the previously unrecognized role of the poly-K in membrane association, may explain the difficulty of demonstrating the requirement of *N*-myristoylation for plasma membrane association in previous reports (Ishitani et al., 2000).

Removal of the poly-K did not interfere with the binding of the SOS3-K/A mutant to SOS2 in the BiFC test. However, the resulting protein complex showed a marked cytosolic localization, similar to that of the SOS3-G2A mutant in complex with SOS2, and in contrast with the wild-type SOS3-SOS2 complex that showed a strong association with the cellular membrane (Figure 8). Moreover, the SOS3-K/A mutant failed to reconstitute a functional SOS module in yeast cells, akin to the SOS3-G2A mutant (Figure 3). Together, these findings suggest that *N*-myristoylation anchors SOS3 to the membrane and that the poly-K track is a structural determinant for the correct positioning of the SOS2-SOS3 complex to the membrane plane for the successful activation of SOS1.

In this work, we have also addressed the long-standing observation that some CBLs enter the nucleus, including SOS3/CBL4 (Batistic et al., 2010). Out of the four CBLs that are known to be *N*-myristoylated and *S*-acylated (Figure 1A), two of them (CLB1 and CBL9) have a selective association to the plasma membrane, whereas the other two (SOS3/CBL4 and CBL5) display nucleo-cytoplasmic distribution when expressed alone as GFP-protein fusions in plant cells (Batistic et al., 2010). However, the fusion of only the N-terminal part of SOS3/CBL4 to GFP (18 first amino acids) directed the recombinant protein to the cell membrane, implying that information for nuclear localization of SOS3 resides downstream the N-terminal domain subjected to the dual lipidation. There are no canonical nuclear localization signals (NLS) in SOS3. Our attempts to identify non-canonical NLS by site-directed mutagenesis have resulted unfruitful, apart from identifying the *S*-acylation at Cys3 as the key feature leading to nuclear entry. However, we found that simultaneously suppressing *N*-myristoylation and *S*-acylation in the double mutant G2A/C3A restored nuclear localization, suggesting again that information for the nuclear localization of SOS3 resides further downstream in the polypeptide. To reconcile these puzzling observations, we suggest that the *S*-acylation of SOS3 may trigger the proteolytic removal of the protein moiety modified with fatty acids. In this scenario, the co-translational *N*-myristoylation dictates the trafficking of SOS3 toward the plasma membrane in a regulated manner (see below) while conditional *S*-acylation re-directs the double acylated protein toward the nucleus. During this process, N-terminal proteolysis dictated by the *S*-acylation status of SOS3 would result in a fully soluble SOS3 protein that could be accumulated in the nucleoplasm owing to intrinsic signals further downstream in the protein. This would be important for the novel function of nuclear localized SOS3 in the chromatin of the gene *CONSTANS*, a key flowering time regulator (Park et al., 2021). The hypothetical proteolytic step is likely to occur at the nuclear envelope or the perinuclear ER since the substrate is the double acylated SOS3 protein. Removal of all acylation signals in the G2A/C3A would mimic the N-terminal processed protein.

**Figure 9:**
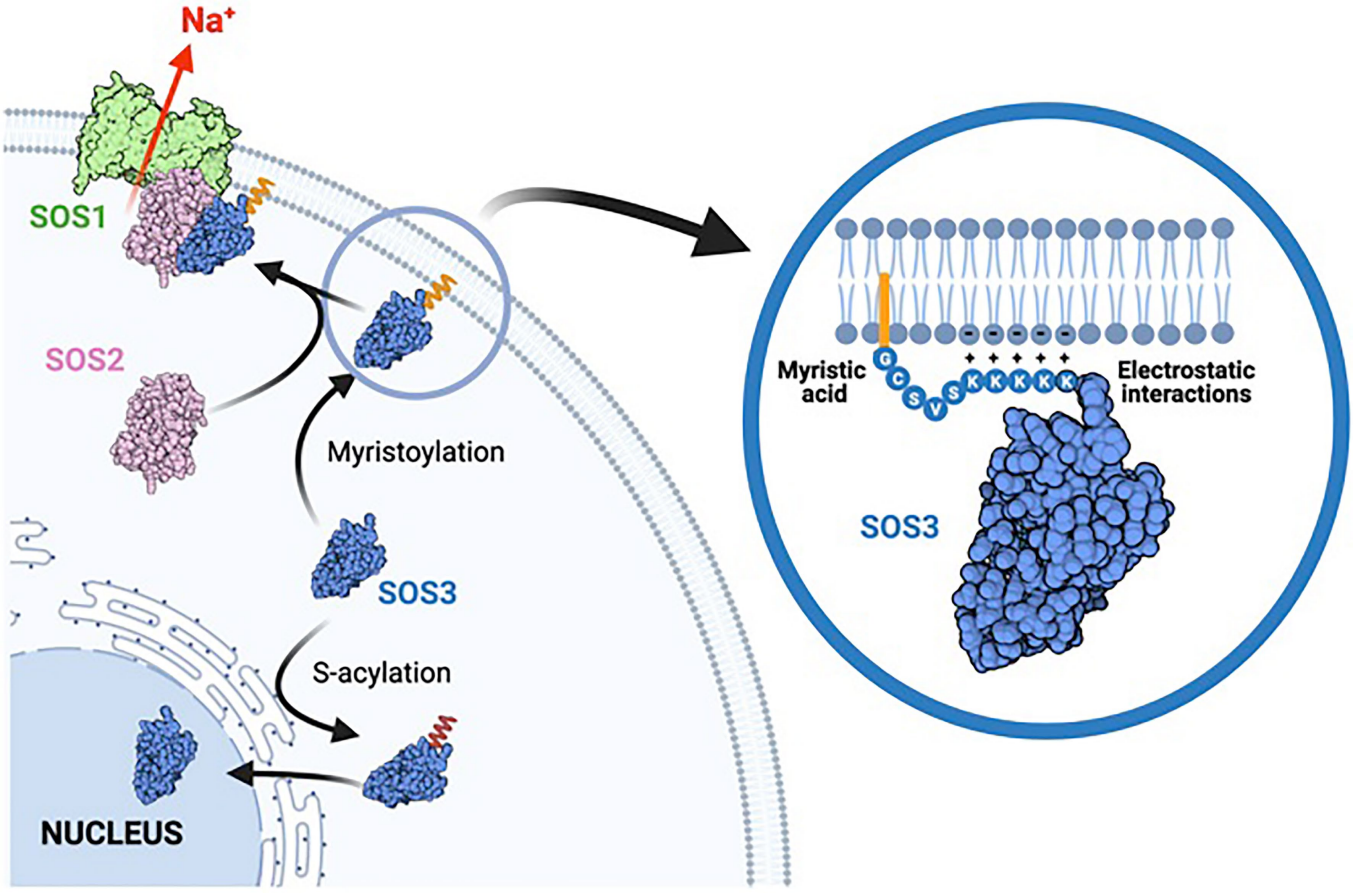
Model of acylation-dependent SOS3 localization. Co-translational *N*-myristoylation of SOS3 results in the recruitment of the SOS2-SOS3 kinase complex to the plasma membrane for the phosphorylation and activation of SOS1 protein, thereby leading to sodium efflux and salt tolerance. The poly-lysine tract adjacent to the acylation sites reinforces the attachment of SOS3 to the plasma membrane by forming salt-bridges with the polar heads of phospholipids. By contrast, S-acylation commands the nuclear trafficking of SOS3 toward the nucleus. Cytosolic SOS3 may be either non-acylated as depicted or have the myristic residue concealed within the protein structure. We suggest that SOS3 is de-acylated when entering the nucleus to remain soluble in the nucleoplasm.

Our results demonstrate that the *S*-acylation of SOS3, which should in theory contribute with additional hydrophobicity to reinforce the membrane attachment of *N*-myristoylated SOS3, actually led to the trafficking of SOS3 toward the nucleus. The non-palmitoylatable mutant SOS3-C3A displayed a bi-partite segregation between the plasma membrane and cytosol (Figures 6, 7), suggesting that either a fraction of the wild-type SOS3 is not *N*-myristoylated or that the *N*-myristoyl group is somehow concealed. Hydrophobic proteins are synthesized in the rough-ER, packaged into vesicles, and transported to the Golgi. However, soluble proteins like SOS3 can be synthesized by cytoplasmic polysomes, thereby escaping *N*-myristoylation by NMTs and remaining cytosolic. Consistent with this, the soluble proteins of *Arabidopsis* that were potential substrates for *N*-myristoylation displayed mixed amino termini *in vivo*, either *N*-myristoylated or keeping a free N-terminal glycine (Castrec et al., 2018). In the alternative scenario, the myristoyl group of SOS3 might not be constantly exposed and reduced to a simple membrane-anchoring role. In recoverin, a CBL-like protein of animals, the myristoyl group can swing between folding inside a hydrophobic protein pocket and extending to the outside upon Ca^2+^ binding in a process denoted as a calcium-myristoyl switch (Tanaka et al., 1995). Calcineurin-homologous proteins (CHP) from animals, which are also closely related to plant CBLs, can associate reversibly to microsomal membranes *via* a calcium-myristoyl switch (Di Sole et al., 2012). We show here that the *N*-myristoylation of SOS3 is neither an impediment nor an aid for nuclear targeting since both the wild-type and the G2A mutant proteins localized to the nucleus (Figures 4, 5). Thus, it appears that wild-type SOS3 is either only partially *N*-myristoylated *in vivo* or able to conceal the myristoyl group, while still functionalizing the *S*-palmitoyl moiety to dictate trafficking toward the nucleus (Figure 8). Our attempts to experimentally determine by protein mass-spectrometry the fraction of SOS3 polypeptides that are *N*-myristoylated, *S*-palmitoylated or keep a free N-terminal glycine have resulted unfruitful, possibly due to the N-terminal poly-K track leading to inconsistent proteolytic digestion and heterogeneous ions hard to identify by proteomic analyses. However, the fact that the SOS2-SOS3 complex observed by BiFC is largely found at the cell surface (Figure 8) suggests that the *N*-myristoyl group of SOS3 is fully displayed upon SOS2 binding to bring the protein kinase complex to the proximity of SOS1 (Figure 9). Last, we have shown that *N*-myristoylation is not a pre-requisite for *S*-acylation since the G2A mutant of SOS3 was still *S*-acylated (Park et al., 2021) and directed toward the nucleus (Figure 5).

Palmitoylation is known to regulate either protein retention or anterograde trafficking at the ER–Golgi, and protein cycling within the endosomal system (Aicart-Ramos et al., 2011). In this regard, it is important to note that the *S*-acylation of SOS3/CBL4 is required to exit the AKT2-CIPK6 complex out of the ER-Golgi and toward the plasma membrane (Held et al., 2011). AKT2 is a K^+^-selective channel operating at the plasma membrane, and CIPK6 is a CBL4-interacting kinase that facilitates the intracellular trafficking of AKT2 by a phosphorylation-independent mechanism that relies on protein–protein interaction. Here, we show the opposite route, in which *S*-acylation of SOS3 results in retrograde trafficking toward the nucleus. Since canonical *N*-myristoylation of SOS3 at Gly2 takes place concurrently with protein synthesis, additional *S*-acylation at Cys3 catalyzed by DHHC-type PATs resident at the ER (Batistic, 2012) could increase membrane association of the dual-acylated protein and retention in the ER membrane. From there, SOS3 would be delivered into the nucleus, likely after the proteolytic removal of the N-terminus.

We have shown that SOS3 *S*-palmitoylation switches the default pathway of *N*-myristoylated SOS3 toward the plasma membrane for the activation of SOS1 and instead promotes the trafficking of SOS3 toward the nucleus (Figure 8). We have recently shown that SOS3 interacts with GIGANTEA (GI; Park et al., 2021), a component of the circadian clock that inputs blue-light signals into the photoperiodic pathway to flowering (Sawa et al., 2007). The SOS3-GI complex is directed toward the nucleus by an unknown mechanism that is strictly dependent on the *S*-acylation of SOS3 at Cys3. In this system, SOS3 functions to regulate flowering time under salinity stress through the transcriptional activation of CONSTANS (CO) and FLOWERING LOCUS T (FT). We envision that upon specific environmental or cellular clues and depending on the interacting partners, *S*-acylated SOS3 may be trafficked to the plasma membrane (Held et al., 2011) or redirected to the perinuclear ER and eventually enter the nucleus (Park et al., 2021). Thus, conditional *S*-acylation of SOS3 serves as a molecular switch that dictates the subcellular distribution of SOS3 and the biological output linked to its activity (Figure 8).

To summarize our conclusions, we provide evidence that *N*-myristoylation of SOS3 at Gly2 together with the adjacent poly-basic domain serve, in response to a salinity-induced Ca^2+^-spike (Koster et al., 2019), to anchor and position the SOS2-SOS3 complex in the plasma membrane and to activate SOS1 for salt tolerance. However, upon unknown input signals, SOS3 becomes *S*-acylated at Cys3 and recruited to the nucleus to regulate other stress responses, such as the fine-tuning of flowering time under stress environments (Park et al., 2021).

## Materials and Methods

### Plasmid Constructs

To express the wild-type and mutants of SOS3 in yeast cells, the wild-type cDNA of *SOS3* was amplified by PCR using the following forward primers 5'-GATCTAGAATGGGCTGCTCTGTATC (for SOS3 wild-type), 5'-GATCTAGAATGGCCTGCTCTGTATC (for the Gly2 to Ala mutant; G2A), 5'-GATCTAGAATGGGCGCCTCTGTATC (for the Cys3 to Ala mutant, C3A), 5'-GATCTAGAATGGCCGCCTCTGTATC (for the double mutation G2A and C3A) and 5'-GACTCTAGAATGGGCTGCTCTGTATCGGCGGCGGCGGCGGCGAATGCAATGCGACCAC (for the poly-K to poly-A; K/A), with the reverse primer 5'-TCTGCGGCCGCGGAAGATACGTTTTGCAA in all amplifications. *Xba*I and *Not*I restrictions sites are underlined in the primer sequences. PCR fragments were digested with *Xba*I and *Not*I, purified and subcloned into a pYPGE15 vector (Brunelli and Pall, 1993) modified to include a 6xHis-tag that was translationally fused to the C-terminal of SOS3. For the Sos Recruitment System (SRS), these *SOS3* constructs were mobilized to vector pADNS in frame with C-terminal hSos (Aronheim, 1997).

To express these SOS3 variants with C-terminal GFP fusions in *Nicotiana benthamiana*, the corresponding *SOS3* cDNAs were transferred to vector pGreen-II with the *CaMV35S* promoter (John Innes Centre). For co-localization with subcellular markers, the nucleo-cytoplasmic mCherry protein in plasmid pGGZ-YL038 (Waadt et al., 2019), and the mRFP-tagged plasma membrane protein LTI6b (plasmid pUBN-mRFP-LTI6b) were used. To generate pUBN-mRFP-LTI6b, an entry clone encoding LTI6b (*At3g05890*) was constructed by PCR amplification of the LTI6b coding sequence from PM-YC3.6-Lti6b (Krebs et al., 2012) using primers 5'-AAAAAGCAGGCTGTATGAGTACAGCCAC-3' and 5'-AGAAAGCTGGGTTCACTTGGTGATG-3' that contained attB1 and attB2 sites, respectively. The gel-purified PCR product was introduced into pDONR221 (ThermoFisher) using BP-clonase II to yield pDONR211-LTI6b. A correct entry clone was verified by sequencing. The LR recombination reaction using LR-clonase II was performed with vectors pUBN-mRFP-DEST (Grefen et al., 2010) and pDONR211-LTI6b to generate pUBN-mRFP-LTI6b. For the BiFC tests, the cDNAs encoding wild-type SOS3 and mutants G2A, C3A y K/A were recovered from pYPGE15 and cloned as *XbaI*/*XhoI* inserts in vector pSPYCE(M) (Waadt et al., 2008) to produce C-terminal fusion to the C-terminal part of YFP. Plasmid pSPYNE(R):SOS2 with an N-terminal fusion of SOS2 to the N-terminal part of YFP was a gift from Prof. J. Kudla (Munster University). For stable expression in *Arabidopsis*, cDNA constructs encoding the wild-type SOS3 and the mutant alleles SOS3-G2A, SOS3-C3A, and SOS3-K/A were transferred to vector pBI321 (Martinez-Atienza et al., 2007) and expressed under the *CaMV35S* promoter.

### Transformation and Growth Conditions

Yeast cells were propagated at 28°C in YPD medium (1% yeast extract, 2% bacto peptone, and 2% glucose) and transformed by the lithium acetate-PEG method (Elble, 1992). Yeast transformants were selected in YNB medium (0.17% yeast nitrogen base without amino acids, 0.5% ammonium sulphate, 2% glucose) supplemented with the corresponding amino acids. Yeast drop-test was used to determine the tolerance of yeast transformants to NaCl in synthetic Arginine-Phosphate (AP) medium (8mM H_3_PO_4_, 10mM L-arginine, 2mM MgSO_4_, 0.2mM CaCl_2_, 1mM KCl, 2% glucose, 1% oligoelements, 1% vitamins, and pH 8.5 with L-arginine; Rodriguez-Navarro and Ramos, 1984). For this, the sodium-sensitive strain YP890 (*MAT alpha, ∆ena1::HIS3::ena4, ∆nha1::LEU2, ∆nhx1::KanMX4, PGK1_PRO_:SOS1::CYC1, ade2, his3, leu2, trp1, can1-100*) was used (Guo et al., 2004). For drop-tests in plates, decimal serial dilutions were made from saturated overnight cultures in YNB (O.D.600~2), and 5μl drops of each dilution were spotted onto selective AP plates supplemented with the NaCl concentrations stated in each experiment. Plates were incubated at 28°C during 3–5days. For the SRS, transformants of the thermosensitive strain *cdc25-2* (*MAT alpha, cdc25-2, ade2, his3, leu2, lys2, trp1, ura3, GAL^+^*) were plated in two plates of YNB without leucine and were incubated at the permissive (25°C) or restrictive (37°C) temperatures. In these experiments, at least three independent yeast transformants for each construct were compared to ascertain coherent phenotypes. Only one representative clone is shown in the figures. All experiments were repeated at least twice.

Plants (*Arabidopsis thaliana* and *N. benthamiana*) were grown in soil in climatically controlled chambers at 25/20±2°C, 40/60% relative humidity, 16/8h day/night cycles and 150–200μmolm^2^s^−1^ PAR. The *Agrobacterium tumefaciens* strain GV3101 was used for all plant transformations. Competent cells were transformed by electroporation and selected in YEP medium (1% yeast extract, 1% bacto peptone, and 0.5% KCl) supplemented with the appropriate antibiotics. The mutant *sos3-1* of *Arabidopsis* (Liu and Zhu, 1998) was transformed by the floral dipping method (Clough and Bent, 1998). Plants were grown under long-day conditions to induce flowering. The first bolts were removed to allow the proliferation of many secondary inflorescences from the rosette’s axillary buds. Completely developed flowers were eliminated during the 3days previous to transformation to decrease the percentage of non-transformed siliques. Plant inflorescences were dipped for 30s in an *A. tumefaciens* suspension containing 5% (w/v) sucrose and 0.02% (v/v) Silwet L-77 as surfactant. After dipping, plants were sealed in plastic bags and transferred to the greenhouse until F1 seeds were harvested. Hygromycin-resistant T1 transgenic plants were selected on Murashige and Skoog (MS) agar medium supplemented with 20μg/ml hygromycin B. Resistant plants were transferred to soil. T2 transgenic plants that showed a 3:1 segregation for the resistant marker were grown on soil in the greenhouse, and T3 seeds were harvested. T3 transgenic plants homozygous for the resistance trait were selected in MS with hygromycin B. Experiments were done with homozygous lines. The *sos3-1* genotype of all transformants was confirmed by diagnostic PCR with the reverse oligo 5’-GTCTTTCCATTCATCTATATCG-3', and the forward oligos 5'-GTTGCTTTCAAGTTACGACAAA-3' and 5'-GTTGCTTTCAAGTTGTACGATT-3 that discriminate for the presence or not of the 9bp deletion corresponding to allele *sos3-1* (Liu and Zhu, 1998). The presence of transgenes was determined with oligos annealing to the *SOS3* and *NPT-II* coding regions.

For the salt-tolerance test, 10-day old *Arabidopsis* seedlings germinated on MS plates were transferred onto fresh plates of LAK medium supplemented with 1mM KCl and the indicated amounts of NaCl. The LAK medium is a Long-Ashton mineral solution modified to be nominally free of Na^+^, K^+^, and NH_4_^+^ and that allows the precise monitoring of tolerance/sensitivity to the Na^+^ ion while minimizing the osmotic effects of high NaCl concentrations commonly used in MS medium (Barragan et al., 2012). Seedlings were incubated vertically at 25°C, 16h/8h light period and 30µE m^−2^s^−1^ PAR for 10–14days. The experiment was repeated twice, with similar outcomes.

### Chemical Analysis of SOS3 Fatty Acid Content

Yeast cells expressing His-tagged SOS3 were grown overnight in 500ml YPD medium at 30°C. Cells were collected by centrifugation and resuspended in buffer A (50mM NaH_2_PO_4_, pH 8.0, and 300mM NaCl) supplemented with 10mM imidazole and 1mM PMSF. The cell suspension was lysed using glass beads following standard protocols. Unbroken cells and debris were discarded after centrifugation at 3,000g for 10min. The supernatant was centrifuged at 20,000g for 30min, and the clarified total protein extract was incubated overnight with 250μl of Ni–NTA resin (Novagen). The resin was packed in a 1ml column (BioRad) and washed four times with buffer A with 20mM imidazole. Finally, bound protein was eluted with buffer A supplemented with 250mM imidazole.

Prior to analyzing the linked fatty acid profile, the purified protein was acidified with 100μl of formic acid and washed three times with *n*-pentane to remove non-covalently bound lipids then fatty acids methyl esters (FAMEs) were prepared. Protein samples were placed in 10ml test tubes with Teflon caps, frozen at −80°C and lyophilized. The dry material was submitted to a combined base-catalyzed and acid-catalyzed hydrolysis and transesterification. Briefly, 0.75ml of 0.5M sodium methoxide (NaMeO) was added, and the mixture was flushed with nitrogen and heated and maintained at 80°C for 2h. After cooling to room temperature, 1.5ml of 3% (v/v) H_2_SO_4_-MeOH was added and subsequently heated at 80°C for 6h. The mixture was left to cool at room temperature, and then 1ml of heptane (containing 0.05% BHT) and 3ml 10% (w/v) were added and mixed vigorously for 1min. The upper organic phase, corresponding to heptane, was collected for GC analysis. FAMEs were separated on a Supelcowax 10 fused silica capillary column (30m length; 0.32mm internal diameter; 0.25μm film thickness; Sigma-Aldrich) by using an Agilent Technologies 7890A gas chromatograph fitted with flame ionization detection, a split/splitless injector, and a 7683B series automatic liquid sampler. Helium was used as carrier gas with a constant linear flow of 1.75ml/min. The injector and detector temperatures were 250°C and 260°C, respectively. The oven temperature program started at 145°C, increasing with a ramp of 15°C/min to 230°C with a 10min hold. Injection volume was 1μl at a split ratio of 1:50. Fatty acids were identified by comparison with known standards.

### Transient Expression in *Nicotiana benthamiana* and Microscopy

*A. tumefaciens* strain GV3101 was transformed by electroporation with constructs for the transient expression of GFP-fused SOS3 proteins and Bimolecular Fluorescence Complementation (BiFC). These transformants were used together with the p19 strain by infiltration of 5–6-week-old *N. benthamiana* leaves after bacterial activation with 150μm acetosyringone. Microscopy analyses and BiFC were performed as in Waadt et al. (2008). The nucleo-cytoplasmic marker mCherry (plasmid pGGZ-YL038) and the plasma membrane marker RFP:LTI6b (plasmid pB7WGR2-UBQ10-mRFP-LTI6b) were used as controls for the co-localization of GFP-tagged SOS3 proteins. *Nicotiana benthamiana* leaf discs were cut 3days after infiltration.

Laser scanning confocal microscopy of SOS3-GFP, LTI6b-RFP, and mCherry fluorescent proteins, and of BiFC with SOS2/SOS3 complexes was done with a FluoView FV1000 Confocal Microscope (Olympus) fitted with multiline-argon and HeNe laser sources, and with UPLSAPO 20×0.75 and 60×1.20 water objectives, and UPLAPO 40×1.00 oil immersion objective. The following combinations of excitation/emission filters were used: 488/510nm for GFP, 515/527nm for YFP, and 543/581nm for LTI6b-RFP. Images were processed with the Olympus FluoView 2.1 software (Olympus). Co-localization of wild-type SOS3-GFP and mutant proteins with mCherry and LTI6B-RFP was also determined with a Leica TCS-SP5II confocal microscope equipped with multiline-argon and DPSS-561 diode laser sources and a Leica HCX PL APO lambda blue 63.0×1.20 UV water immersion objective. Excitation/emission settings were 488/500–540nm for GFP, 561/580–610nm for LTI6B-RFP, and 561/610–640 for mCherry. For co-localization experiments, fluorescence was captured simultaneously in the green and red channels. Fluorescence intensity in cell transects was measured with Fiji software. All microscopy experiments were repeated at least twice and multiple cells (*n*>10) were observed in each one. Images shown in microscopy figures are representative single Z-sections of 512×512 or 1024×1024 pixels.

## Data Availability Statement

The raw data supporting the conclusions of this article will be made available by the authors, without undue reservation.

## Author Contributions

IV, EG, CT, IM, and FQ conducted the functional analyses in yeast, *Arabidopsis* transformation, and salinity tolerance tests. IV, EG, RC, AL, and ZA performed the microscopy analyses of protein localization and BiFC. DH-M and FQ determined the fatty acid profile of SOS3. IV, KS, JP, and FQ designed and supervised the research. JP and FQ wrote the manuscript. All authors checked and approved the manuscript.

## Funding

This work was supported by grant no. P18-RT-3991 from Consejería de Conocimiento, Investigación y Universidad, Junta de Andalucía (to FQ), grant nos. RTI2018-094027-B-I00 (to JP) and PID2019-109664RB-100 (to FQ) from Agencia Estatal de Investigacion (AEI-MCIU, Spain), co-financed by the European Regional Development Fund.

## Conflict of Interest

The authors declare that the research was conducted in the absence of any commercial or financial relationships that could be construed as a potential conflict of interest.

## Publisher’s Note

All claims expressed in this article are solely those of the authors and do not necessarily represent those of their affiliated organizations, or those of the publisher, the editors and the reviewers. Any product that may be evaluated in this article, or claim that may be made by its manufacturer, is not guaranteed or endorsed by the publisher.
